# Specificity of the T Cell Response to Protein Biopharmaceuticals

**DOI:** 10.3389/fimmu.2020.01550

**Published:** 2020-07-22

**Authors:** Sylvain Meunier, Marie de Bourayne, Moustafa Hamze, Aurélien Azam, Evelyne Correia, Catherine Menier, Bernard Maillère

**Affiliations:** Université Paris-Saclay, CEA, INRAE, Département Médicaments et Technologies pour la Santé, SIMoS, Gif-sur-Yvette, France

**Keywords:** T cell, biopharmaceuticals, therapeutic antibody, therapeutic protein, immunogenicity, epitope, T cell selection, tolerance

## Abstract

The anti-drug antibody (ADA) response is an undesired humoral response raised against protein biopharmaceuticals (BPs) which can dramatically disturb their therapeutic properties. One particularity of the ADA response resides in the nature of the immunogens, which are usually human(ized) proteins and are therefore expected to be tolerated. CD4 T cells initiate, maintain and regulate the ADA response and are therefore key players of this immune response. Over the last decade, advances have been made in characterizing the T cell responses developed by patients treated with BPs. Epitope specificity and phenotypes of BP-specific T cells have been reported and highlight the effector and regulatory roles of T cells in the ADA response. BP-specific T cell responses are assessed in healthy subjects to anticipate the immunogenicity of BP prior to their testing in clinical trials. Immunogenicity prediction, also called preclinical immunogenicity assessment, aims at identifying immunogenic BPs and immunogenic BP sequences before any BP injection in humans. All of the approaches that have been developed to date rely on the detection of BP-specific T cells in donors who have never been exposed to BPs. The number of BP-specific T cells circulating in the blood of these donors is therefore limited. T cell assays using cells collected from healthy donors might reveal the weak tolerance induced by BPs, whose endogenous form is expressed at a low level. These BPs have a complete human sequence, but the level of their endogenous form appears insufficient to promote the negative selection of autoreactive T cell clones. Multiple T cell epitopes have also been identified in therapeutic antibodies and some other BPs. The pattern of identified T cell epitopes differs across the antibodies, notwithstanding their humanized, human or chimeric nature. However, in all antibodies, the non-germline amino acid sequences mainly found in the CDRs appear to be the main driver of immunogenicity, provided they can be presented by HLA class II molecules. Considering the fact that the BP field is expanding to include new formats and gene and cell therapies, we face new challenges in understanding and mastering the immunogenicity of new biological products.

## Introduction

Protein biopharmaceuticals (BPs) have revolutionized the treatment of many diseases and their share of the worldwide drug market continues to grow. There are three main categories of protein BPs, if we exclude recombinant vaccines and allergen-specific immunotherapy. Antibodies and molecules composed of antibody fragments are the most important category in terms of the number of BPs and market share. Hormones, growth factors and cytokines define a category of recombinant human proteins with an endogenous counterpart generally circulating at low concentration. The third category comprises replacement proteins such as clotting factors or lysosomal enzymes, which are used to restore partial or complete genetic deficiencies. All BPs exhibit marked specificity for their target and low toxicity. However, BPs may be immunogenic and promote immune responses against themselves. In particular, anti-drug antibodies (ADAs) may be produced by patients following infusion of BPs and may disturb the pharmacokinetics of BPs, neutralize their therapeutic activities or induce allergic or autoimmune symptoms, depending on the category of BPs.

Several therapeutic antibodies have been found to induce neutralizing antibodies, as illustrated by the anti-TNFα antibodies infliximab and adalimumab (1, 2). Infusion and allergic reactions have also been reported for infliximab (3). ADAs raised against the second category of BPs, which includes recombinant hormones (insulin, H2-relaxin) (4, 5), growth factors [erythropoietin (Epo), GM-CSF] (6, 7) and cytokines (IFN-β, IFN-α, IL-2) (8), might be neutralizing as well. An additional risk exists for this category, owing to the existence of endogenous forms. ADAs raised against the recombinant protein can also neutralize the protein produced by the patients and could affect critically important functions of the endogenous protein. Pure red cell aplasia (PRCA) is a rare autoimmune disease characterized by rapidly progressive severe anemia resulting from the disappearance of erythroid precursor cells in the bone marrow. PRCA can be mediated by specific ADAs induced by injections of recombinant human Epo, which has a fully human peptide sequence (6). Neutralizing antibodies are also an important issue for replacement proteins, as alternative therapies to infusion with the protein remain limited and, in some cases, may be reserved mostly for patients with severe deficiencies. Neutralizing antibodies to factor VIII (FVIII) (often called inhibitors in hemophilia papers) are found in 30% of patients with severe hemophilia A and cause severe clinical complications (9). Patients with mild or moderate severity hemophilia A have a lower incidence of inhibitors, which is in part due to residual FVIII, which actively tolerizes to the drug., but also to their lack of or lower exposure to therapeutic FVIII. Those who have received intensive FVIII therapy have an inhibitor incidence approaching that of severe hemophilia A patients (9, 10). The incidence of inhibitors of factor IX is lower in hemophilia B (11).

All BPs have either entirely or partially humanized sequences, but humanization does not guarantee a complete lack of immune response as illustrated by the examples above. Immune tolerance is firstly a consequence of B and T cell selection during their ontogeny. B and T cell repertoires are counter-selected by self-proteins in the bone marrow (12) and the thymus (13), respectively. However, the level of endogenous counterparts of BPs, which are expected to promote tolerance, might completely differ across BP categories and patients and therefore lead to variable levels of drug tolerance. IgG1 antibodies are present in the plasma at a concentration of approximately 9 mg/mL. The sequences of the constant parts of human IgG1 antibodies are therefore present as high levels of self-proteins. In contrast, VH or VL sequences are expressed at variable and lower levels (14), while all the sequences resulting from gene rearrangement and somatic mutations are not present in the human proteome. The second category corresponds to self-proteins present at low levels and the replacement proteins forming the third category might have different levels of endogenous counterpart. In patients with complete deficiency, there is no endogenous counterpart and the replacement protein is a foreign protein for the patients. In contrast, a mutated form circulates at variable concentration (also called cross-reacting material CRM) in patients with mild or moderately severe disease (15). Completely different physiological conditions therefore shape the repertoire of BP-specific CD4 T cells across BP categories and are expected to influence the initial BP-specific T cell frequency, peptide specificity and T cell expansion upon BP infusion. As CD4 T cells participate in the initiation and control of immune responses to either foreign or self-molecules, all these parameters should impact initiation and regulation of the ADA response and therefore help to understand and anticipate ADA onset. The objective of this paper is to review recent developments in the understanding the T cell response to BPs, with a main focus on mechanisms controlling peptide specificity, T cell selection and regulation.

## Basic Mechanisms of the Antigen-Specific CD4 T Cell Response

**Figure 1:**
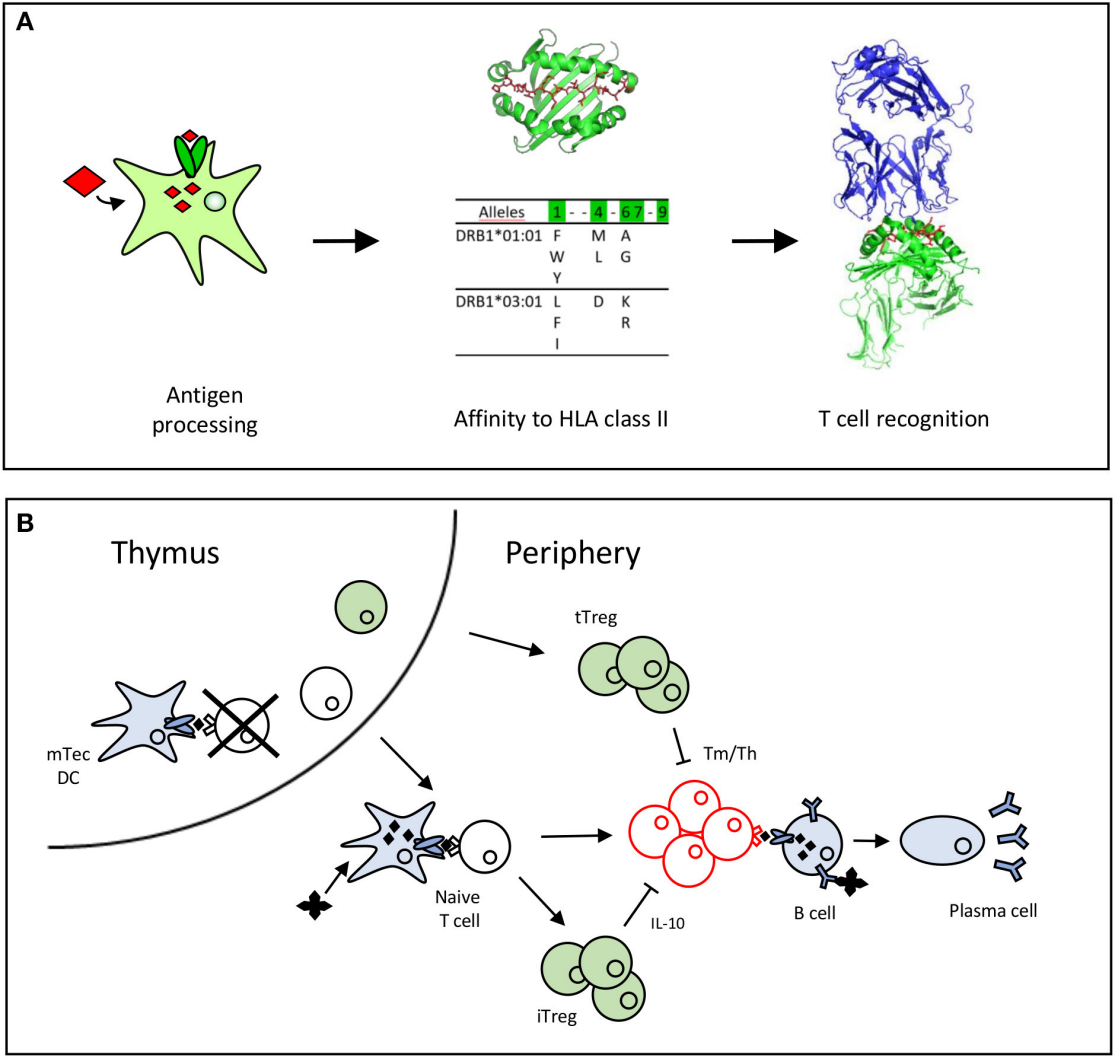
Antigen recognition by T cells leads to T cell selection and expansion. **(A)** Peptide requirements for T cell recognition (19). A T cell epitope is processed by the dendritic cell, binds to HLA class II molecules (16, 20) and is recognized by the TCR (21). **(B)** T cells are selected positively and negatively by recognition of self-peptides in the thymus. In the periphery, the naïve T cells recognize antigenic peptides to expand and differentiate into memory or effector T cells (Tm/Teff). Thymic T cells (tTregs) are committed in the thymus to regulatory T cells, while induced regulatory T cells (iTreg), that secrete IL-10 are differentiated from the pool of naïve cells in the periphery.

CD4 T cells recognize antigens as peptides displayed by HLA class II molecules at the surface of dendritic cells (DCs). These peptides are produced by proteolysis of antigens by cathepsins in the endosomal compartments and possess appropriate amino acid sequences (also called binding motifs) (16, 17) that allow their binding to the polymorphic HLA class II molecules (18). As the polymorphic residues are mainly present in the peptide binding site of the HLA class II molecules, the bound peptides might be restricted to particular HLA class II allotypes or, in contrast, might benefit from shared binding properties across allotypes to bind multiple HLA class II molecules (16, 17). Some of the presented peptides are recognized by CD4 T cells (Figure 1A). CD4 T cells express at their surface a specific receptor (TCR), which selectively recognizes peptides anchored to the HLA molecules across the multitude of displayed peptides. This selectivity comes from the wide diversity of TCRs generated by the random and imprecise rearrangements of the V and J segments of the TCR alpha and V, D, and J segments of the TCR beta genes (22). Pioneering estimates of the diversity of the TCR repertoire suggest a lower limit estimate of 1 million different TCR beta genes (23, 24), but more recent investigations using high-throughput sequencing propose minimal estimates of 100 million unique TCRβ sequences (25). This TCR repertoire is further shaped by self-peptides in the thymus (13), where many autoreactive T cells are deleted by apoptosis (Figure 1B). T cells leaving the thymus have not been in contact with the antigen in the periphery and are called naïve T cells. Some T cells specific for self-peptides might also be committed to thymic regulatory cells (tTregs) in the thymus (Figure 1B). The diversity of TCRs renders each antigen-specific naïve T clonotype very rare among the whole population of T cells, but at varying frequencies depending on the nature of the antigens (26) (Figure 1B). Frequencies of T cells specific for a single epitope evaluated using MHC class II tetramers (27, 28) or limiting dilution conditions vary in the range of 10^−7^ to 10^−5^ for tumor (29, 30) or foreign antigens (27, 28, 31). These frequencies are higher for full-length proteins, as exemplified by KLH or protective anthrax antigen (32, 33), but are expected to be low for recombinant forms of self-proteins owing to negative selection of T cells in the thymus (13). Upon encountering antigens in the body, specific T cell clones are activated in the lymphoid organs and expanded over the other T cells. This expansion is in the range of 10- to 1,000-fold for CD4 cells (27) (Figure 1B). Interestingly, the final frequencies of antigen-specific memory cells are correlated with the frequencies of precursors in the naïve repertoire, as shown in mice and humans using MHC class II tetramers (26–28). Finally, during the expansion, activated CD4 T cells transform into memory cells with helper functions. Some of the expanded T cells might also transform into induced regulatory T cells (iTreg), which are mainly characterized by their capacity to release immunosuppressive cytokines such IL-10 (Figure 1B).

## T Cell Dependence of the ADA Response

Whether ADA induction is T cell-dependent is not an obvious question as pioneering work on the T cell response to FVIII using synthetic peptides did not reveal particular differences between hemophilia A patients who did or did not develop neutralizing antibodies (34–36). It thus appears important to clarify the necessary role of the T cell response in the ADA response for the three categories of BPs, as T cell dependence validates the interest of focusing on the T cell response to provide insights into immunogenicity.

From these pioneering studies, the T cell response to FVIII did not appear to be linked to the ADA response, but multiple lines of evidence later pointed to T cell dependence (34–36). Anti-FVIII antibodies are of the IgG isotype and contain somatic mutations in their variable domains indicating that they have been produced by B cells that have undergone isotype switching and affinity maturation (37), both of which are T cell-dependent. Many studies have reported the T cell response in patients with mild/moderate (38–40) or severe hemophilia A (40, 41) who developed neutralizing ADAs. Most patients with mild or moderate hemophilia A circulate a point-mutated, partially disabled FVIII protein and require only rare infusions of therapeutic FVIII, indicative of they are less likely to develop an ADA response (38, 39). However, intensive treatment to support surgery or treat major bleeds, or accumulated occasional exposures over a lifetime, increase their risk of immunogenicity substantially (10). Analysis of the epitope-specificity of the T and B cell response in these patients showed that both FVIII T cells and antibodies might be raised against the mutated epitopes, suggesting a tight regulation of the specificity of FVIII-specific T cells (38, 39, 42). An interesting observation is that low CD4 T cells resulting from HIV infection of hemophilia A patients was associated with less anti-FVIII antibodies, suggesting an important role of T cell help (43). In line with this finding in HIV infection, mouse models using FVIII-deficient mice also indicated that ADA induction against FVIII requires CD4 T cell activation (44–46).

For the second category of BPs, i.e., hormones, growth factors, and cytokines, a higher T cell response was found in ADA+ patients treated with IFN-beta as compared to ADA– patients and healthy donors (47). The T cell dependence of IFN-beta immunogenicity was strengthened by HLA association of the ADA response to IFN-beta (48, 49) and was also shown in IFN-beta transgenic mice (50). Patients treated with recombinant human erythropoietin and who developed PRCA showed a clear T cell response to recombinant human erythropoietin, in contrast to ADA– patients (51).

Finally, for the category constituted by the therapeutic antibodies, Vultaggio et al. compared the T cell response to anti-TNFα infliximab in 32 ADA+ patients, 39 ADA– patients and 10 healthy donors (52). Infliximab-specific cell proliferation was detected mainly with T cells collected from ADA+ patients, especially those who developed hypersensitivity reactions, as compared to T cells collected from ADA- patients or healthy donors. The T cell response in ADA+ patients treated with infliximab or rituximab was also observed by others (53) and the ADA response to infliximab was found to be associated with HLA-DRB1^*^03 (54). Isolation of human monoclonal antibodies (mAbs) raised against adalimumab (55) and natalizumab (56) confirmed the T cell dependence of their *in vivo* generation, their sequences containing multiple somatic mutations. Anti-natalizumab mAbs were isolated from donors who developed a T cell response (56). Altogether CD4 T cell response appears as a requisite to mount a ADA response for the three BP categories.

## T Cell Response to BPs Using Cells Collected From Healthy Donors With a View to Predicting Immunogenicity

A prerequisite for the generation of a CD4 T cell response to BP is the presence of T cells in the T cell repertoire that recognize epitopes within the BP. *In vitro* stimulation assays using T cells from healthy unexposed subjects are generally used to assess the potential reactivity to BP. This is in contrast to investigations of T cell responses against foreign proteins, whose T cell response is mainly investigated using donors who have already mounted an immune response to the antigens. This difference impacts both the methodologies and the outcomes of the T cell assays applied to BPs. Indeed, owing to the risk that immunogenicity issues stop the clinical development of new products, an important request from pharmaceutical companies is anticipation of these issues by selecting the least immunogenic BPs across the BP candidates at the early stages of drug development. Generally, drug selection is driven by preclinical studies carried out in animal models. However, animal models are not considered as good models for predicting the immunogenicity of BPs in humans, the humanized proteins being recognized as non-self in animals (57). As CD4 T cells are involved in the initiation of the immune responses, T cell assays using cells collected from healthy donors have been developed to evaluate whether BPs could prime a new T cell response *in vitro* (58–61). These T cell assays evaluate whether T cells circulating in the blood of healthy donors can recognize the BPs. They are clearly different from assays that are done with cells collected from patients developing an ADA response. T cell assays using cells collected from healthy donors provide an estimate of the number of T cells prone to react to BP recognition in healthy donors, who serve as estimators of the number of T cells in the patients before BP injection. Therefore, they do not therefore directly predict immunogenicity but reveal a “potential of response,” which is one of the main factors contributing to immunogenicity (57). Multiple formats of T cell assays are used to predict BP immunogenicity. Cells introduced in the assay can be either PBMCs (PBMC assay) or a co-culture of autologous DCs and T cells (DC:T cell assay) (58, 60, 61). Assays also differ by the number of *in vitro* stimulations with either BPs (59) or mitogenic molecules (33) and by the readout used to characterize T cell specificity (mainly CFSE, 3H-thymidine incorporation or ELISPOT). T cell assays are validated by comparing BPs known to be either immunogenic or non-immunogenic in humans (58–61), assuming that the response in *in vitro* experiments correlates with an immune response in patients. Because of the low frequency of naïve BP-specific T cells in healthy donors (26), we developed a T cell assay relying on a long-term culture phase to enrich the cell culture in specific T cells (T cell amplification assay) (59, 62). This was adapted from assays developed to identify tumor antigens (63, 64). In this assay, antibodies known to be immunogenic in multiple treated patients, such as adalimumab (2), infliximab (1, 65), and rituximab (66, 67), generate a higher T cell response (59, 68) than poorly immunogenic antibodies such as trastuzumab (69) and secukinumab (70) or fusion protein (etanercept) (71). Finally, T cell assays using healthy donors have provided most of the data on the T cell response to BPs (58–61, 68). Many T cell epitopes have also been identified using these assays, as described in the following sections.

## mAbs-Specific CD4 T Cell Epitopes and Their Relationship With Thymic Selection

**Figure 2:**
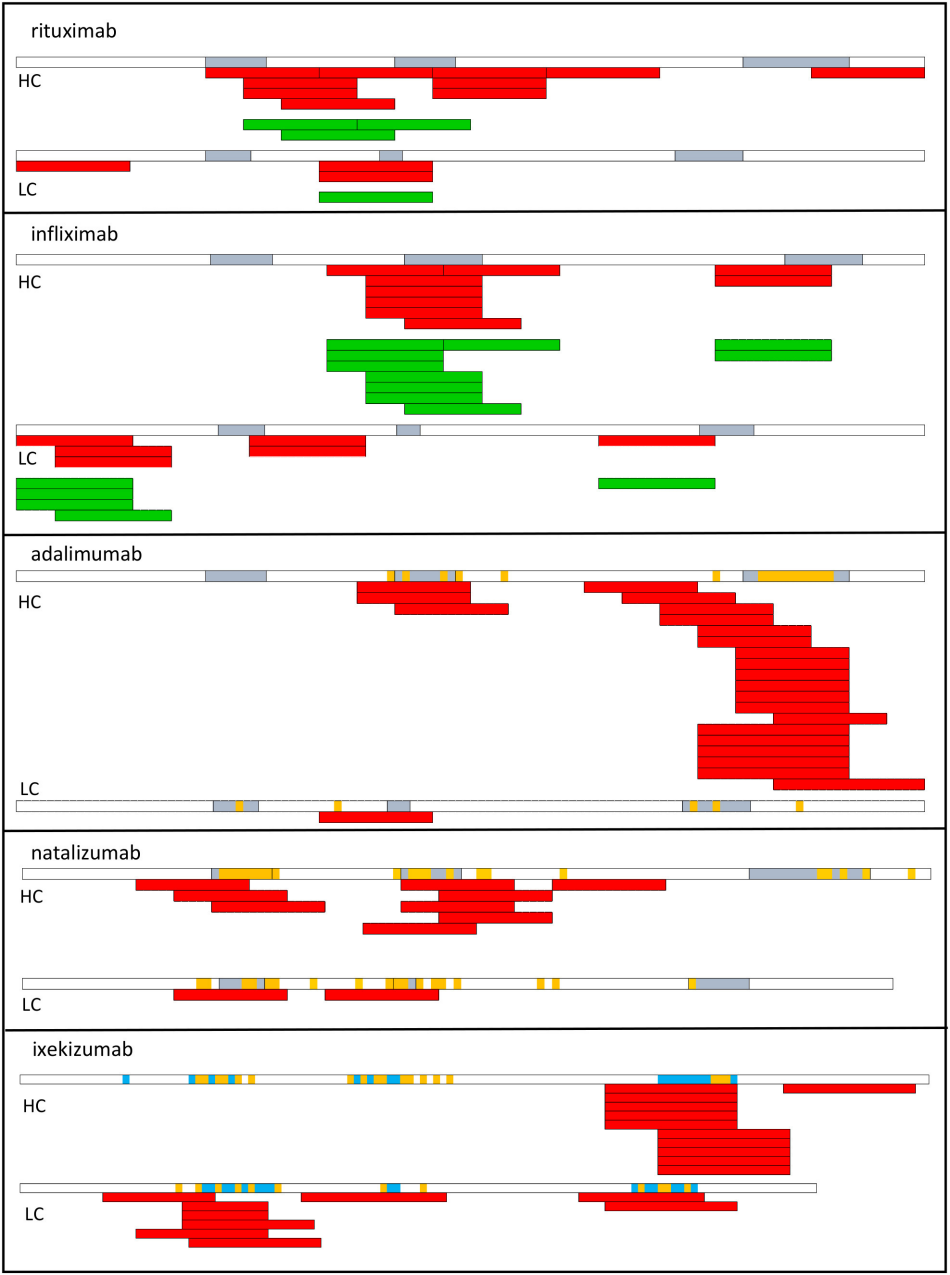
T cell epitopes of therapeutic antibodies. T cell epitopes have been identified from T cells collected from healthy donors (red) or from patients who develop an ADA response (green) to rituximab, infliximab (53), adalimumab, natalizumab (72), and ixekizumab (73). Each bar corresponds to an individual response. CDR regions are shown in blue. Amino acids in orange correspond to mutations with respect to the best-fitting germline sequence. HC, heavy chain; LC, light chain.

**Figure 3:**
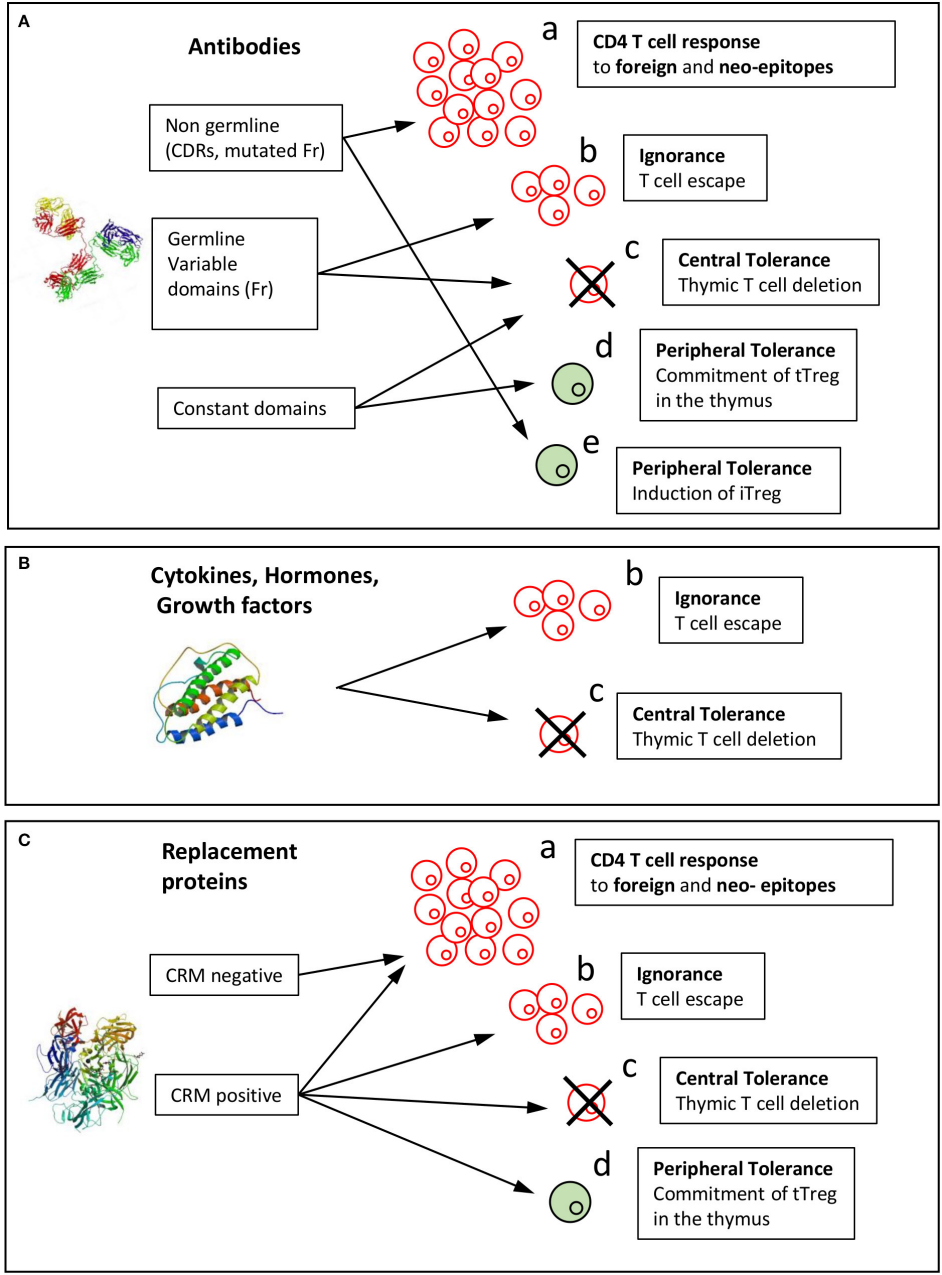
Mechanisms of induction of the T cell response or tolerance to biopharmaceuticals. Immunological mechanisms involved in the immunogenicity of BPs are reported for the three categories of BPs: **(A)** antibody, **(B)** cytokines, hormones, growth factors, **(C)** replacement proteins. Biopharmaceuticals might be recognized by T cells as foreign in gene-deficient patients or as mutated sequences with respect to the human germline sequences (neoepitopes) (a). Non-mutated sequences might (b) stimulate T cells that have escaped thymic selection or might (c) lead to T cell tolerance by deletion of T cells in the thymus or (d) induction of thymic Tregs (tTregs). (e) Peripheral tolerance could be provided by induced Tregs (iTregs). Structure of a full-length antibody (Pdb: 21GF), human Epo (Pdb: 1BUY), and human FVIII (Pdb: 3CDZ).

Because of HLA polymorphism, CD4 T cell epitopes of BPs are expected to vary from one donor to another as a function of their HLA allotypes, but also to be shared by multiple donors owing to common binding specificities of the HLA class II molecules (17). The location of BP-specific T cell epitopes is precious information in understanding which parts of the molecules contribute to their immunogenicity (Figure 2), but also which mechanisms take part in this response. In fact, immunological mechanisms leading to the T cell response to mAbs differ between regions mutated with respect to the germline sequences, non-mutated regions of the variable domains and constant regions (Figure 3A).

T cell epitopes have been identified in multiple therapeutic antibodies, including infliximab (53), rituximab (53), adalimumab (72), natalizumab (72), ixekizumab (73) by deriving CD4 T cell lines from cells collected from healthy donors (Figure 2). The location of CD4 T cell epitopes was found to be very specific for each therapeutic antibody (Figure 2). Two-thirds of the identified T cell epitopes of infliximab and rituximab participated to the T cell response mounted in patients, who developed an ADA response (53). In another study, the T cell response to infliximab in patients treated with infliximab appeared broader (74). To provide insights on the mechanisms leading to the T cell specificity, affinity of the mAb peptides for HLA-DR molecules, which are common worldwide was evaluated and the mAb peptides presented by HLA-DR molecules of mAb-loaded dendritic cells were identified. mAb-specific T cell epitopes are often found to bind to HLA-DR molecules with good affinity (53, 72). For example, HCDR3 of adalimumab is a hot-spot of good binders to HLA-DR molecules (72) and contains the vast majority of identified T cell epitopes. T cell epitopes are often among the peptides identified by MHC-associated peptide proteomics (MAPPs) assay. This assay consists in loading immature monocyte-derived DCs from healthy human donors with mAb, isolating HLA-DR associated peptides from mAb-loaded DCs and identifying them by liquid chromatography-mass spectrometry (53, 72, 73, 75). Importantly, regions mutated with respect to the germline sequences and hence mainly the CDR regions appear as the main regions hosting T cell epitopes, which are therefore recognized as neoepitopes (Figure 3a). Studies in mice have demonstrated that somatic hypermutations create CD4 T cell epitopes in the V regions (Figure 3a), although germline sequences do not elicit a CD4 T cell response (76). Germline sequences lead to central deletion of the specific CD4 T cell precursors in the thymus (77, 78), possibly through their presentation by thymic B cells (79) (Figure 3c). The CDR3 sequences result from the junction of V(D)J segments and TdT-catalyzed addition of nucleotides, and they therefore differ largely from germline sequences. Multiple T cell epitopes from adalimumab and ixekizumab overlap HCDR3 (Figure 3a). In CDR1 and CDR2 of the VH and VL chains of an antibody, somatic mutations are introduced during affinity maturation and seem to generate multiple epitopes of natalizumab and ixekizumab (Figure 3a). T cell clones derived from two multiple sclerosis patients who developed an ADA response specific for natalizumab were found to react with one CD4 T cell epitope overlapping the LCDR2 region, only (56). This epitope was also identified in a study performed with cells collected from healthy donors (72). Interestingly, a T cell response targeting a single epitope can suffice to mount an ADA response (56).

However, some T cells recognizing non-mutated parts of the mAb could escape thymic selection, as shown in a mouse model (78) (Figure 3b). Three T cell epitopes of adalimumab, one of natalizumab and one of ixekizumab do not contain somatic mutations with respect to the germline sequences (Figure 2) (72, 73), as evaluated by two alignment methods (80, 81). The best-fitting germline sequence of adalimumab VH is the gene segment VH3.9 (81). It is a poorly expressed germline gene segment in the human B cell repertoire (14) and hence could give rise to a partial escape of adalimumab-specific CD4 T cells from the thymus (78) (Figure 3b). However, these T cell epitopes contribute only little to the mAb-specific T cell response (Figure 2).

In addition to the T cell epitopes identified in the variable domains of therapeutic mAbs, T cell epitopes deriving from constant regions of IgG (82) and from human serum albumin (83) were found to activate tTreg cells and have been called Tregitopes. Indeed, some precursor T cells egressing from the thymus are committed to T cells with immunosuppressive function and characterized by the CD4+CD25+FoxP3+ phenotype (84). Autoreactive thymic regulatory T cells (tTregs) are positively selected on endogenous self-antigens expressed in the thymus. This selection occurs in a highly specific manner through TCR interaction of high affinity for their cognate antigen. Constant parts of immunoglobulin IgG1 and serum albumin are both highly abundant proteins and should therefore be presented as antigenic peptides by MHC II molecules expressed by medullary thymic epithelial cells (mTECs) in the thymus. Presentation of these peptides might lead to deletion of effector CD4 T cells (Figure 3c), which explains why CD4 T cell epitopes are not found in the constant parts of IgG, and also to selection of specific tTregs (Figure 3d). These Tregitopes have been shown to suppress multiple kinds of immune responses, including humoral (82, 85) and cellular (86), but were also shown to be poorly active in this regard in an alternative study (87). No BP-specific tTregs have been cloned and characterized yet and the mechanism of suppression remains to be determined. Together, the T cell repertoire specific for therapeutic mAbs appears to be shaped by creation of neoepitopes for mutated regions with respect to the germline sequences, T cell escape (also called ignorance see below) and deletion in the thymus for non-mutated regions of the variable domains and T cell deletion or tTreg generation for constant regions (Figure 3A).

## CD4 T Cell Repertoire of Hormones, Growth Factors, and Cytokines

Mutations cannot be the only driving force of BP immunogenicity, as immunogenic hormones, growth factors and cytokines that form the second BP category are not mutated. Cytokines such as IFN-b (88), IFN-α (89), and IL-2 (90) and growth factors such as GM-CSF (7) are all known to elicit an antibody response in some patients, albeit to various degrees. *In vivo* expression of the endogenous counterpart of immunogenic BPs of the second category might not be sufficient to completely deplete the specific T cells in the thymus, leading to immunological ignorance, as named by Ohashi et al. (91) (Figure 3b). This immunological mechanism has been demonstrated in transgenic mice expressing a model antigen in the periphery (91). In these mice, antigen-specific T cells escape from thymic selection as the model antigen is not expressed in the thymus. T cells, however, do not react to the antigen expressed in the periphery, owing to the lack of appropriate signals of T cell activation.

Similarly, expression of the endogenous counterpart of the second BP category might be not sufficient to completely deplete the specific T cells in the thymus. T cells escape from thymic selection (Figure 3b), although they are specific for self-sequences and not neoepitopes (Figure 3a). A specific and functional T cell repertoire is therefore available to react to the corresponding BPs, but is not activated at the steady state. As an example, a high frequency of Epo-specific T cells was found in the blood of many healthy donors (62, 92). In most patients, infusion of correctly formulated recombinant Epo is well-tolerated and probably did not activate Epo-specific T cells, in the absence of co-stimulatory signals. However, altered HSA-free Epo batches might contain Epo aggregates, micelles and leachates from the syringe stopper (93), which might favor DC maturation and antigen capture by the DCs (94) and thereby provide T cell activation signals (51). Another example of immunogenic BP of the second category is relaxin. Relaxin is a two-chain peptide hormone structurally related to insulin with anti-inflammatory activity, but its injection in humans led to the production of anti-relaxin antibodies in multiple patients (4). In agreement with its clinical immunogenicity, relaxin elicits a strong *in vitro* T cell response from cells collected from healthy donors (95).

In agreement with the concept of antigen ignorance (91) (Figure 3b), T cell epitopes have been identified in Epo (92), IFN-b (47), and relaxin (95), using synthetic peptides bearing unmodified human sequences. Given that the sequences of the BP are identical to those of the endogenous forms, these findings confirm that BP can be immunogenic without alteration of their sequence (Figure 3b), e.g., by chemical (96) or post-translational modifications to create neo-epitopes (Figure 3a). In fact, to our knowledge, T cell neoepitopes of the second category of BPs have not yet been identified.

One important consideration in anticipating the tolerance or ignorance of self-sequences might be direct evaluation of their expression in the thymus. Epo and IFN-b do not seem to be expressed in the thymus, in agreement with their capacity to mount a T cell response (47, 51, 62, 92). In contrast, the thymic expression of relaxin has been reported (95), but it is unknown whether relaxin is expressed by mTECs to promote T cell selection. To our knowledge, insulin is the only hormone known to induce central tolerance, as depletion of insulin expression in mTECs has been shown to induce spontaneous anti-insulin autoimmunity in mice (97) (Figure 3d). In the case of insulin, a particularity is, that in some cases, insulin is given to diabetic patients with autoantibodies against self-proteins and peptides including insulin itself (98, 99). Hence autoantibodies to insulin may be preexisting, i.e., present before the first injection of insulin. Multiple studies have identified proinsulin and not only insulin as the main target of autoreactive CD4 T cells, many clones being specific for the C-peptide human proinsulin (100–102). Mechanisms of insulin immunogenicity are therefore not representative of the other immunogenic hormones, but suggest that expression of self-antigens in mTec might be investigated to define the immunogenicity risk of therapeutic proteins. In summary T cells specific for cetagory including hormones, growth factors and cytokines are therefore either produced by ignorance or deleted by central tolerance (Figure 3B).

## CD4 T Cell Epitopes of Replacement Proteins, Illustrated by FVIII

The third category of BPs comprises replacement proteins such as clotting factors or lysosomal enzymes. To our knowledge, there are no T cell epitopes identified in lysosomal enzymes such as α- glycosidase (Pompe disease), α-galactosidase (Fabry disease), or in factor IX (hemophilia B) in humans, but only in mice (103). In contrast, multiple studies have sought to identify CD4 T cell epitopes of FVIII from severe hemophilia A patients (41, 104), mild/moderate hemophilia A patients (38, 39, 105, 106) and FVIII-deficient humanized mice (46).

T cell reactivity against FVIII appears very complex and depends on the residual amount of endogenous FVIII. The grade of hemophilia A from severe to mild is related to the remaining level of FVIII function. Deficiencies range from complete lack of circulating FVIII, which results in the most severe hemophilia A cases, to an altered function due to missense mutations, small insertions or deletions. ADA incidence varies from 30% in severe hemophilia A patients to 5–20% in mild/moderate hemophilia A patients and may be linked to the remaining amount of FVIII (CRM+) and may not be related to its functionality (107). Hemophilia B patients have a reduced risk of neutralizing antibodies, probably because patients produce a circulating and dysfunctional factor IX (CRM+) (11).

In severe hemophilia A patients, partial or complete lack of FVIII (CRM–) renders the infused FVIII a foreign molecule (Figure 3a). In line with the large size of FVIII, multiple potential epitopes have been found to be displayed by HLA class II molecules (108, 109) and are across all FVIII domains (108–110). The number of presented peptides is diminished by the association of FVIII with von Willebrand factor (110), which limits the uptake of FVIII by DCs (111). In FVIII-deficient HLA-DR15-transgenic mice, which are designed to mimic severe hemophilia A, the T cell response is supported by 8 dominant epitopes, which exhibit a good affinity for different HLA-DR molecules. T cell epitopes identified in FVIII (36, 38, 39, 106) are some of the presented peptides (108) but all the presented FVIII peptides did not elicit a T cell response as already observed for therapeutic mAbs (53, 72). The T cell response in severe hemophilia A patients may appear to focus on only one high-avidity epitope, when assessed with HLA-Class II tetramers (41), which is at variance with studies using T cell proliferation assays suggesting a much broader T cell response to FVIII (34).

In mild/moderate hemophilia A patients, the remaining material of FVIII circulating in the blood might exert selective pressure on the CD4 T cells in the thymus (Figure 3c). This is supported by observation patients with mild-moderate hemophilia that carry a mutation. CD4 T cell clones isolated from these patients target the mutated epitope (i.e., a neoepitope) (Figure 3a), suggesting that T cells reactive to the non-mutated variant were negatively selected. Both the epitope with the R2150H mutation and the non-mutated counterpart bind with high affinity to multiple HLA-DR molecules, the mutation supposedly being in tight contact with the TCR (38). The epitope with the A2201P mutation (39, 105) was the same as that found in severe hemophilia A patients (41). In contrast to the mutated regions, the remaining parts of FVIII did not give rise to specific T cells in the mild/moderate hemophilia A patients and appeared to be tolerated (Figure 3c).

Strikingly, induction of central tolerance (Figure 3c) by endogenous FVIII does not seem to be important in healthy donors. Indeed, a large repertoire of FVIII-specific CD4 T cells is found in the blood of healthy donors (112), suggesting that these cells escape negative thymic selection (Figure 3b). Half of the FVIII-specific T cells were naïve or memory T cells and the frequency of memory cells remained low as compared to memory cells specific for foreign antigens (112).

This repertoire of FVIII-reactive T cells might also trigger the naturally-occurring anti-FVIII Abs found in many healthy subjects and maybe even for the onset of acquired hemophilia (113). Owing to the higher incidence of ADAs in severe hemophilia A patients as compared to mild/moderate hemophilia A patients, the different level of FVIII deficiency might enlarge the FVIII-specific CD4 T cell repertoire from the already large repertoire found in healthy donors, by expanding T cells of higher avidity for their cognate antigen, a concept demonstrated for a tumor antigen in mice (114). Alternatively, the loss of FVIII expression in hemophilia A patients could be accompanied by a deficiency of thymic regulatory T cells (tTregs). Indeed, absence of cognate antigen has been shown to lead to a severe loss of tTreg positive selection (115). Deficiency in expression of FVIII in hemophilia A patients may therefore impact FVIII-specific tTreg selection (Figure 3d). But to date, the role of tTreg cells in the FVIII T cell response has only been assessed by removing CD4+ CD25+ T cells from T cell proliferation assays (105) or by adoptive transfer of tTregs into mice challenged with FVIII plasmid (116). No FVIII peptides have yet been characterized for tTreg recognition. Further studies using samples from mild/moderate or severe hemophilia A patients or healthy donors are required to provide a more complete picture of the FVIII T cell epitopes and how they entail or prevent ADA onset. Nevertheless, how the T cell repertoire is shaped in CRM– and CRM+ patients appears to involve multiple immunological mechanisms.

## Role of Subsets of BP-Specific CD4 T Cells in the Induction and Regulation of the ADA Response

Different subsets of T cells participate in the initiation and regulation of many adaptive immune responses, their frequencies, phenotypes, onset and persistence might be a source of understanding, as already observed for vaccines (117) and allergen immunotherapies (118, 119). To date, the phenotypes of T cells specific for BPs in treated patients have been investigated in few studies, mainly in the context of mAbs (52, 53, 120). Overall, the data suggest that the ADA response might be controlled by the balance between antibody-inducing and antibody-suppressing cytokines. Although ADA follow-up is requested by the authorities during clinical trials, characterization of the T cell response is not mandatory, but recommended. Ethical issues might also limit the collection of blood samples, especially as in most genetic deficiencies, including hemophilia and lysosomal diseases, the patients might be very young. As mentioned above, effector T cells specific for infliximab (52, 53, 120), rituximab (53), and natalizumab (56) collected from patients who developed an ADA response were revealed by proliferation assays or secretion assays of inflammatory cytokines. Individual patterns of the cytokines including IFN-γ, IL-13 (120), IL-5, and IL-17 (53) were found across the patients. IL-13 appeared to be associated with detection of specific IgE (52). T cell clones specific for infliximab were found to be of TH1, TH2, or TH0 cells (120) and should provide help to the B cells to produce ADA. By contrast, many of the T cell clones (120) or bulk T cells (53, 120) also secreted IL-10, an immunosuppressive cytokine, which is known to suppress T cell proliferation and IgG secretion (121) (Figure 3e). IL-10-secreting T cell clones specific for infliximab inhibited the proliferation of co-cultured effector T clones also specific for infliximab in an IL-10-specific manner (120). Interestingly, longitudinal analysis of the infliximab-specific T cell response highlighted the upregulation of IL-10 throughout the treatment, while IFN-y was mainly expressed at the first infusion. Patients who developed ADAs produced little IL-10 at the beginning of the treatment and exhibited low IL-10/IFN-γ ratios (74). IL-10 was therefore found to be a cornerstone of ADA regulation. IL-10-producing cells are induced by infusion with the therapeutic antibody (Figure 3e) and differ from tTreg cells (Figure 3d), which are directly committed to regulatory T cells in the thymus (84). Besides IL-10 production (121), induced Treg cells (iTregs) (Figure 3e) might acquire a Foxp3+ CD25+ CD4+ phenotype (120) similar to that of tTregs (Figure 3d), but they share the same CD4 T cell epitopes with effector CD4 T cells, as they are produced from the same precursor cells, as shown for allergen immunotherapy (122). In studies of the T cell response to infliximab (52, 120), almost all patients generated an IL-10 response, including patients who did not generate ADA (ADA-). It is generally thought that ADA- patients did not develop any immune response against the BP. As shown in this study, they might develop a suppressive T cell response, which hampers the ADA response. Isolated immunomonitoring of the ADA response, while neglecting the T cell response, may therefore underestimate the number of patients who generate an immune response to infliximab (1, 65). Further investigations are required to generalize these observations to other anti-TNF therapeutic antibodies or other BPs. In conclusion, detailed analyses of the T cell response to BPs are insufficient in number, but the existing studies are important in deciphering a part of the mechanisms of immunogenicity in patients treated with BPs.

## Perspectives

In the last decade, our understanding of the T cell response to BPs has benefited from findings from various immunology-related fields including vaccinology and cancer immunology. T cell assays relying on the generation of T cell lines have been adapted from experiments done to identify tumor T cell epitopes (63). Prediction of T cell epitopes is done using algorithms trained with foreign sequences, especially from bacterial and viral antigens (123). The next decade may benefit from methods developed to investigate the T cell response in healthy donors and in patients, from the identification of their own T cell epitopes, and from pioneering work on T cells in patients treated with BPs. It is important to extend studies to many more BPs, as the paucity of clinical data is hampering progress. Little attention has also been paid to understanding how the T cell response is triggered. A soluble molecule generally leads to immune tolerance rather than immune response (124) and needs adjuvant activity to switch on the response (125). It is unclear how BPs acquire this ability to prime the T cell response. Identified hints are the capacity to aggregate (126), which may occur *in vivo* (127), to contain trace levels of impurities (128), to form immune complexes (129), and to interact with immune cells (130). Moreover, patient- treatment- or disease-specific factors may also have to be considered. An important opportunity provided by T cell epitope identification is to de-immunize BPs by removing T cell epitopes from their sequences (131–133). The challenge of de-immunization is to take into account the wide diversity of HLA in humans and to maintain BP functionality. Multiple molecules have already been engineered including enzymes (132), FVIII (134), and mAbs (56, 133), but to our knowledge none has yet been challenged in a clinical setting. Finally, multiple other BPs different from proteins, such as advanced therapeutic medicinal products, are being actively developed and studied in terms of immunogenicity issues. Pre-existing immunity to recombinant adeno-associated virus vectors or lentiviral vectors compromises the efficacy of gene therapy, while *de novo* antibody and cytotoxic CD8 T cell responses could eliminate cells transfected with the therapeutic products (135). Pre-existing immunity has also been demonstrated for the bacterial CAS9 protein and could dramatically limit genome editing tools such as CRISPR/Cas9 technology (136). All these new therapeutic approaches might benefit from already off-the-shelf technologies to investigate the T cell response and from recent advances in single-cell analysis (137) and next-generation sequencing (138).

## Author Contributions

SM, MH, MB, AA, EC, CM, and BM wrote the manuscript and gave final approval of the version to be published. All authors contributed to the article and approved the submitted version.

## Conflict of Interest

The authors declare that the research was conducted in the absence of any commercial or financial relationships that could be construed as a potential conflict of interest.

## Abbreviations

ADA: anti-drug antibody
CDR: complementarity determining region
BP: biopharmaceuticals
Epo: erythropoietin
DCs: dendritic cells
HLA: human leukocyte antigen
HC: heavy chain
HSA: human serum albumin
IFN-γ: interferon-γ
KLH: keyhole limpet hemocyanin
LC: light chain
MAPPs: MHC-associated peptide proteomics
MS: mass spectrometry
PBMCs: peripheral blood mononuclear cells.

## References

[1] Wolbink GJ Vis M Lems W Voskuyl AE de Groot E Nurmohamed MT. Development of antiinfliximab antibodies and relationship to clinical response in patients with rheumatoid arthritis. Arthritis Rheum. (2006) 54:711–5. 10.1002/art.21671 16508927

[2] Bartelds GM Wijbrandts CA Nurmohamed MT Stapel S Lems WF Aarden L. Clinical response to adalimumab: relationship to anti-adalimumab antibodies and serum adalimumab concentrations in rheumatoid arthritis. Ann Rheum Dis. (2007) 66:921–6. 10.1136/ard.2006.065615 17301106 PMC1955110

[3] Vultaggio A Matucci A Nencini F Pratesi S Parronchi P Rossi O. Anti-infliximab IgE and non-IgE antibodies and induction of infusion-related severe anaphylactic reactions. Allergy. (2010) 65:657–61. 10.1111/j.1398-9995.2009.02280.x 19951375

[4] Seibold JR Korn JH Simms R Clements PJ Moreland LW Mayes MD. Recombinant human relaxin in the treatment of scleroderma. A randomized, double-blind, placebo-controlled trial. Ann Intern Med. (2000) 132:871–9. 10.7326/0003-4819-132-11-200006060-00004 10836913

[5] Chen JW Frystyk J Lauritzen T Christiansen JS. Impact of insulin antibodies on insulin aspart pharmacokinetics and pharmacodynamics after 12-week treatment with multiple daily injections of biphasic insulin aspart 30 in patients with type 1 diabetes. Eur J Endocrinol. (2005) 153:907–13. 10.1530/eje.1.02021 16322398

[6] Casadevall N Dupuy E Molho-Sabatier P Tobelem G Varet B Mayeux P. Autoantibodies against erythropoietin in a patient with pure red-cell aplasia. N Engl J Med. (1996) 334:630–3. 10.1056/NEJM199603073341004 8592526

[7] Wadhwa M Skog AL Bird C Ragnhammar P Lilljefors M Gaines-Das R. Immunogenicity of granulocyte-macrophage colony-stimulating factor (GM-CSF) products in patients undergoing combination therapy with GM-CSF. Clin Cancer Res. (1999) 5:1353–61. 10389919

[8] Meager A Wadhwa M Dilger P Bird C Thorpe R Newsom-Davis J. Anti-cytokine autoantibodies in autoimmunity: preponderance of neutralizing autoantibodies against interferon-alpha, interferon-omega and interleukin-12 in patients with thymoma and/or myasthenia gravis. Clin Exp Immunol. (2003) 132:128–36. 10.1046/j.1365-2249.2003.02113.x 12653847 PMC1808678

[9] Peyvandi F Mannucci PM Palla R Rosendaal FR. SIPPET: methodology, analysis and generalizability. Haemophilia. (2017) 23:353–61. 10.1111/hae.13203 28306186

[10] Abdi A Linari S Pieri L Voorberg J Fijnvandraat K Castaman G. Inhibitors in nonsevere hemophilia a: what is known and searching for the unknown. Semin Thromb Hemost. (2018) 44:568–77. 10.1055/s-0037-1621717 29439277

[11] Goodeve AC. Hemophilia B: molecular pathogenesis and mutation analysis. J Thromb Haemost. (2015) 13:1184–95. 10.1111/jth.12958 25851415 PMC4496316

[12] Rose NR. Negative selection, epitope mimicry and autoimmunity. Curr Opin Immunol. (2017) 49:51–5. 10.1016/j.coi.2017.08.014 29102863

[13] Klein L Kyewski B Allen PM Hogquist KA. Positive and negative selection of the T cell repertoire: what thymocytes see. (and don't see). Nat Rev Immunol. (2014) 14:377–91. 10.1038/nri3667 24830344 PMC4757912

[14] Mroczek ES Ippolito GC Rogosch T Hoi KH Hwangpo TA Brand MG. Differences in the composition of the human antibody repertoire by B cell subsets in the blood. Front Immunol. (2014) 5:96. 10.3389/fimmu.2014.00096 24678310 PMC3958703

[15] McGinniss MJ Kazazian HH Jr. Hoyer LW Bi L Inaba H Antonarakis SE. Spectrum of mutations in CRM-positive and CRM-reduced hemophilia A. Genomics. (1993) 15:392–8. 10.1006/geno.1993.1073 8449505

[16] Rammensee HG Friede T Stevanoviic S. MHC ligands and peptide motifs: first listing. Immunogenetics. (1995) 41:178–228. 10.1007/BF00172063 7890324

[17] Sturniolo T Bono E Ding J Raddrizzani L Tuereci O Sahin U. Generation of tissue-specific and promiscuous HLA ligand databases using DNA microarrays and virtual HLA class II matrices [see comments]. Nat Biotechnol. (1999) 17:555–61. 10.1038/9858 10385319

[18] Roche PA Furuta K. The ins and outs of MHC class II-mediated antigen processing and presentation. Nat Rev Immunol. (2015) 15:203–16. 10.1038/nri3818 25720354 PMC6314495

[19] Adorini L Appella E Doria G Nagy ZA. Mechanisms influencing the immunodominance of T cell determinants. J Exp Med. (1988) 168:2091–104. 10.1084/jem.168.6.2091 2462005 PMC2189152

[20] Stern LJ Brown JH Jardetzky TS Gorga JC Urban RG Strominger JL. Crystal structure of the human class II MHC protein HLA-DR1 complexed with an influenza virus peptide. Nature. (1994) 368:215–21. 10.1038/368215a0 8145819

[21] Hennecke J Wiley DC. Structure of a complex of the human alpha/beta T cell receptor (TCR) HA1.7, influenza hemagglutinin peptide, and major histocompatibility complex class II molecule, HLA-DR4. (DRA^*^0101 and DRB1^*^0401): insight into TCR cross-restriction and alloreactivity. J Exp Med. (2002) 195:571–81. 10.1084/jem.20011194 11877480 PMC2193773

[22] Cobb RM Oestreich KJ Osipovich OA Oltz EM. Accessibility control of V(D)J recombination. Adv Immunol. (2006) 91:45–109. 10.1016/S0065-2776(06)91002-5 16938538

[23] Arstila TP Casrouge A Baron V Even J Kanellopoulos J Kourilsky P. A direct estimate of the human alphabeta T cell receptor diversity. Science. (1999) 286:958–61. 10.1126/science.286.5441.958 10542151

[24] Arstila TP Casrouge A Baron V Even J Kanellopoulos J Kourilsky P. Diversity of human alpha beta T cell receptors. Science. (2000) 288:1135. 10.1126/science.288.5469.1135a 10841721

[25] Qi Q Liu Y Cheng Y Glanville J Zhang D Lee JY. Diversity and clonal selection in the human T-cell repertoire. Proc Natl Acad Sci USA. (2014) 111:13139–44. 10.1073/pnas.1409155111 25157137 PMC4246948

[26] Jenkins MK Moon JJ. The role of naive T cell precursor frequency and recruitment in dictating immune response magnitude. J Immunol. (2012) 188:4135–40. 10.4049/jimmunol.1102661 22517866 PMC3334329

[27] Moon JJ Chu HH Pepper M McSorley SJ Jameson SC Kedl RM. Naive CD4(+) T cell frequency varies for different epitopes and predicts repertoire diversity and response magnitude. Immunity. (2007) 27:203–13. 10.1016/j.immuni.2007.07.007 17707129 PMC2200089

[28] Kwok WW Tan V Gillette L Littell CT Soltis MA LaFond RB. Frequency of epitope-specific naive CD4(+) T cells correlates with immunodominance in the human memory repertoire. J Immunol. (2012) 188:2537–44. 10.4049/jimmunol.1102190 22327072 PMC3997369

[29] Valmori D Souleimanian NE Hesdorffer CS Old LJ Ayyoub M. Quantitative and qualitative assessment of circulating NY-ESO-1 specific CD4(+) T cells in cancer-free individuals. Clin Immunol. (2005) 117:161–7. 10.1016/j.clim.2005.07.004 16103015

[30] Zhang Y Sun Z Nicolay H Meyer RG Renkvist N Stroobant V. Monitoring of anti-vaccine CD4 T cell frequencies in melanoma patients vaccinated with a MAGE-3 protein. J Immunol. (2005) 174:2404–11. 10.4049/jimmunol.174.4.2404 15699177

[31] Castelli FA Szely N Olivain A Casartelli N Grygar C Schneider A. Hierarchy of CD4 T cell epitopes of the ANRS Lipo5 synthetic vaccine relies on the frequencies of pre-existing peptide-specific T cells in healthy donors. J Immunol. (2013) 190:5757–63. 10.4049/jimmunol.1300145 23636059

[32] Gebel HM Scott JR Parvin CA Rodey GE. *In vitro* immunization to KLH. II Limiting dilution analysis of antigen-reactive cells in primary and secondary culture. J Immunol. (1983) 130:29–32. 6183352

[33] Geiger R Duhen T Lanzavecchia A Sallusto F. Human naive and memory CD4+ T cell repertoires specific for naturally processed antigens analyzed using libraries of amplified T cells. J Exp Med. (2009) 206:1525–34. 10.1084/jem.20090504 19564353 PMC2715094

[34] Reding MT Wu H Krampf M Okita DK Diethelm-Okita BM Christie BA. Sensitization of CD4+ T cells to coagulation factor VIII: response in congenital and acquired hemophilia patients and in healthy subjects. Thromb Haemost. (2000) 84:643–52. 10.1055/s-0037-1614081 11057864

[35] Reding MT Okita DK Diethelm-Okita BM Anderson TA Conti-Fine BM. Human CD4+ T-cell epitope repertoire on the C2 domain of coagulation factor VIII. J Thromb Haemost. (2003) 1:1777–84. 10.1046/j.1538-7836.2003.00251.x 12911593

[36] Reding MT Okita DK Diethelm-Okita BM Anderson TA Conti-Fine BM. Epitope repertoire of human CD4(+) T cells on the A3 domain of coagulation factor VIII. J Thromb Haemost. (2004) 2:1385–94. 10.1111/j.1538-7836.2004.00850.x 15304045

[37] Jacquemin MG Desqueper BG Benhida A Vander Elst L Hoylaerts MF Bakkus M. Mechanism and kinetics of factor VIII inactivation: study with an IgG4 monoclonal antibody derived from a hemophilia A patient with inhibitor. Blood. (1998) 92:496–506. 10.1182/blood.V92.2.496 9657749

[38] Jacquemin M Vantomme V Buhot C Lavend'homme R Burny W Demotte N. CD4+ T-cell clones specific for wild-type factor VIII: a molecular mechanism responsible for a higher incidence of inhibitor formation in mild/moderate hemophilia A. Blood. (2003) 101:1351–8. 10.1182/blood-2002-05-1369 12393451

[39] Ettinger RA James EA Kwok WW Thompson AR Pratt KP. HLA-DR-restricted T-cell responses to factor VIII epitopes in a mild haemophilia A family with missense substitution A2201P. Haemophilia. (2010) 16:44–55. 10.1111/j.1365-2516.2008.01905.x 20536985 PMC2885051

[40] Jacquemin M Saint-Remy JM. T cell response to FVIII. Cell Immunol. (2016) 301:8–11. 10.1016/j.cellimm.2015.09.007 26435345

[41] Ettinger RA Paz P James EA Gunasekera D Aswad F Thompson AR. T cells from hemophilia A subjects recognize the same HLA-restricted FVIII epitope with a narrow TCR repertoire. Blood. (2016) 128:2043–54. 10.1182/blood-2015-11-682468 27471234 PMC5073183

[42] Gilles JG Lavend'homme R Peerlinck K Jacquemin MG Hoylaerts M Jorieux S. Some factor VIII (FVIII) inhibitors recognise a FVIII epitope(s) that is present only on FVIII-vWF complexes. Thromb Haemost. (1999) 82:40–45. 10.1055/s-0037-1614627 10456452

[43] Bray GL Kroner BL Arkin S Aledort LW Hilgartner MW Eyster ME. Loss of high-responder inhibitors in patients with severe hemophilia A and human immunodeficiency virus type 1 infection: a report from the Multi-Center Hemophilia Cohort Study. Am J Hematol. (1993) 42:375–9. 10.1002/ajh.2830420408 8493988

[44] Qian J Borovok M Bi L Kazazian HHJr Hoyer LW. Inhibitor antibody development and T cell response to human factor VIII in murine hemophilia A. Thromb Haemost. (1999) 81:240–4. 10.1055/s-0037-1614450 10063999

[45] Pratt KP Qian J Ellaban E Okita DK Diethelm-Okita BM Conti-Fine B. Immunodominant T-cell epitopes in the factor VIII C2 domain are located within an inhibitory antibody binding site. Thromb Haemost. (2004) 92:522–8. 10.1160/TH03-12-0755 15351848

[46] Steinitz KN van Helden PM Binder B Wraith DC Unterthurner S Hermann C. CD4+ T-cell epitopes associated with antibody responses after intravenously and subcutaneously applied human FVIII in humanized hemophilic E17 HLA-DRB1^*^1501 mice. Blood. (2012) 119:4073–82. 10.1182/blood-2011-08-374645 22394599 PMC3986681

[47] Kalluri SR Grummel V Hracsko Z Pongratz V Pernpeintner V Gasperi C. Interferon-beta specific T cells are associated with the development of neutralizing antibodies in interferon-beta treated multiple sclerosis patients. J Autoimmun. (2018) 88:83–90. 10.1016/j.jaut.2017.10.003 29066027

[48] Barbosa MD Vielmetter J Chu S Smith DD Jacinto J. Clinical link between MHC class II haplotype and interferon-beta (IFN-beta) immunogenicity. Clin Immunol. (2006) 118:42–50. 10.1016/j.clim.2005.08.017 16260183

[49] Hoffmann S Cepok S Grummel V Lehmann-Horn K Hackermuller J Stadler PF. HLA-DRB1^*^0401 and HLA-DRB1^*^0408 are strongly associated with the development of antibodies against interferon-beta therapy in multiple sclerosis. Am J Hum Genet. (2008) 83:219–27. 10.1016/j.ajhg.2008.07.006 18656179 PMC2495071

[50] Kijanka G Sauerborn M Boon L Schellekens H Brinks V. Development of ADA against recombinant human interferon beta in immune tolerant mice requires rapid recruitment of CD4(+) T cells, induces formation of germinal centers but lacks susceptibility for (most) adjuvants. J Pharm Sci. (2015) 104:396–406. 10.1002/jps.24170 25219665

[51] Rubic-Schneider T Kuwana M Christen B Assenmacher M Hainzl O Zimmermann F. T-cell assays confirm immunogenicity of tungsten-induced erythropoietin aggregates associated with pure red cell aplasia. Blood Adv. (2017) 1:367–79. 10.1182/bloodadvances.2016001842 29296951 PMC5738985

[52] Vultaggio A Petroni G Pratesi S Nencini F Cammelli D Milla M. Circulating T cells to infliximab are mainly detectable in treated patients developing anti-drug antibodies and hypersensitivity reactions. Clin Exp Immunol. (2016) 186:364–72. 10.1111/cei.12858 27569750 PMC5108070

[53] Hamze M Meunier S Karle A Gdoura A Goudet A Szely N. Characterization of CD4 T cell epitopes of infliximab and rituximab identified from healthy donors. Front Immunol. (2017) 8:500. 10.3389/fimmu.2017.00500 28529511 PMC5418239

[54] Billiet T Vande Casteele N Van Stappen T Princen F Singh S Gils A. Immunogenicity to infliximab is associated with HLA-DRB1. Gut. (2015) 64:1344–5. 10.1136/gutjnl-2015-309698 25876612

[55] van Schouwenburg PA Kruithof S Votsmeier C van Schie K Hart MH de Jong RN. Functional analysis of the anti-adalimumab response using patient-derived monoclonal antibodies. J Biol Chem. (2014) 289:34482–8. 10.1074/jbc.M114.615500 25326381 PMC4263856

[56] Cassotta A Mikol V Bertrand T Pouzieux S Le Parc J Ferrari P. A single T cell epitope drives the neutralizing anti-drug antibody response to natalizumab in multiple sclerosis patients. Nat Med. (2019) 25:1402–7. 10.1038/s41591-019-0568-2 31501610 PMC6795539

[57] EMA. Guideline on Immunogenicity Assessment of Biotechnology-Derived Therapeutic Proteins. EMEA/CHMP/BMWP/14327/2006 (2006).

[58] Baker MP Jones TD. Identification and removal of immunogenicity in therapeutic proteins. Curr Opin Drug Discov Devel. (2007) 10:219–27. 17436557

[59] Delluc S Ravot G Maillere B. Quantitative analysis of the CD4 T-cell repertoire specific to therapeutic antibodies in healthy donors. Faseb J. (2011) 25:2040–8. 10.1096/fj.10-173872 21368101

[60] Jawa V Cousens LP Awwad M Wakshull E Kropshofer H De Groot AS. T-cell dependent immunogenicity of protein therapeutics: preclinical assessment and mitigation. Clin Immunol. (2013) 149:534–55. 10.1016/j.clim.2013.09.006 24263283

[61] Schultz HS Reedtz-Runge SL Backstrom BT Lamberth K Pedersen CR Kvarnhammar AM. Quantitative analysis of the CD4+ T cell response to therapeutic antibodies in healthy donors using a novel T cell:PBMC assay. PLoS ONE. (2017) 12:e0178544. 10.1371/journal.pone.0178544 28562666 PMC5451071

[62] Delluc S Ravot G Maillere B. Quantification of the preexisting CD4 T-cell repertoire specific for human erythropoietin reveals its immunogenicity potential. Blood. (2010) 116:4542–5. 10.1182/blood-2010-04-280875 20702780

[63] Chaux P Vantomme V Stroobant V Thielemans K Corthals J Luiten R. Identification of MAGE-3 epitopes presented by HLA-DR molecules to CD4(+) T lymphocytes. J Exp Med. (1999) 189:767–78. 10.1084/jem.189.5.767 10049940 PMC2192951

[64] Zarour HM Maillere B Brusic V Coval K Williams E Pouvelle-Moratille S. NY-ESO-1 119-143 is a promiscuous major histocompatibility complex class II T-helper epitope recognized by Th1- and Th2-type tumor-reactive CD4+ T cells. Cancer Res. (2002) 62:213–8. 11782380

[65] Baert F Noman M Vermeire S Van Assche G D' Haens G Carbonez A. Influence of immunogenicity on the long-term efficacy of infliximab in Crohn's disease. N Engl J Med. (2003) 348:601–8. 10.1056/NEJMoa020888 12584368

[66] Pijpe J van Imhoff GW Spijkervet FK Roodenburg JL Wolbink GJ Mansour K. Rituximab treatment in patients with primary Sjogren's syndrome: an open-label phase II study. Arthritis Rheum. (2005) 52:2740–50. 10.1002/art.21260 16142737

[67] Smith KG Jones RB Burns SM Jayne DR. Long-term comparison of rituximab treatment for refractory systemic lupus erythematosus and vasculitis: remission, relapse, and re-treatment. Arthritis Rheum. (2006) 54:2970–82. 10.1002/art.22046 16947528

[68] Spindeldreher S Maillere B Correia E Tenon M Karle A Jarvis P Secukinumab demonstrates significantly lower immunogenicity potential compared to ixekizumab. Dermatol Ther. (2018) 8:57–68. 10.1007/s13555-018-0220-y PMC5825325 29392570

[69] Van Walle I Gansemans Y Parren PW Stas P Lasters I. Immunogenicity screening in protein drug development. Expert Opin Biol Ther. (2007) 7:405–18. 10.1517/14712598.7.3.405 17309332

[70] Blauvelt A Prinz JC Gottlieb AB Kingo K Sofen H Ruer-Mulard M. Secukinumab administration by pre-filled syringe: efficacy, safety and usability results from a randomized controlled trial in psoriasis. (FEATURE). Br J Dermatol. (2015) 172:484–93. 10.1111/bjd.13348 25132411

[71] Dore RK Mathews S Schechtman J Surbeck W Mandel D Patel A. The immunogenicity, safety, and efficacy of etanercept liquid administered once weekly in patients with rheumatoid arthritis. Clin Exp Rheumatol. (2007) 25:40–6. 17417989

[72] Meunier S Hamze M Karle A de Bourayne M Gdoura A Spindeldreher S. Impact of human sequences in variable domains of therapeutic antibodies on the location of CD4 T-cell epitopes. Cell Mol Immunol. (2019) 17:656–8. 10.1038/s41423-019-0304-3 31659246 PMC7264247

[73] Spindeldreher S Karle A Correia E Tenon M Gottlieb S Huber T. T cell epitope mapping of secukinumab and ixekizumab in healthy donors. MAbs. (2020) 12:1707418. 10.1080/19420862.2019.1707418 31924123 PMC8648323

[74] Pratesi S Nencini F Grosso F Dies L Bormioli S Cammelli D. T cell response to infliximab in exposed patients: a longitudinal analysis. Front Immunol. (2018) 9:3113. 10.3389/fimmu.2018.03113 30687319 PMC6336713

[75] Sekiguchi N Kubo C Takahashi A Muraoka K Takeiri A Ito S. MHC-associated peptide proteomics enabling highly sensitive detection of immunogenic sequences for the development of therapeutic antibodies with low immunogenicity. MAbs. (2018) 10:1168–81. 10.1080/19420862.2018.1518888 30199322 PMC6284561

[76] Eyerman MC Zhang X Wysocki LJ. T cell recognition and tolerance of antibody diversity. J Immunol. (1996) 157:1037–46. 8757607

[77] Snyder CM Aviszus K Heiser RA Tonkin DR Guth AM Wysocki LJ. Activation and tolerance in CD4(+) T cells reactive to an immunoglobulin variable region. J Exp Med. (2004) 200:1–11. 10.1084/jem.20031234 15226360 PMC2213315

[78] Detanico T Heiser RA Aviszus K Bonorino C Wysocki LJ. Self-tolerance checkpoints in CD4 T cells specific for a peptide derived from the B cell antigen receptor. J Immunol. (2011) 187:82–91. 10.4049/jimmunol.1002287 21622865 PMC3124280

[79] Perera J Meng L Meng F Huang H. Autoreactive thymic B cells are efficient antigen-presenting cells of cognate self-antigens for T cell negative selection. Proc Natl Acad Sci USA. (2013) 110:17011–6. 10.1073/pnas.1313001110 24082098 PMC3801014

[80] Lazar GA Desjarlais JR Jacinto J Karki S Hammond PW. A molecular immunology approach to antibody humanization and functional optimization. Mol Immunol. (2007) 44:1986–98. 10.1016/j.molimm.2006.09.029 17079018

[81] Lefranc MP Giudicelli V Duroux P Jabado-Michaloud J Folch G Aouinti S. IMGT(R), the international ImMunoGeneTics information system(R) 25 years on. Nucleic Acids Res. (2015) 43:D413–22. 10.1093/nar/gku1056 25378316 PMC4383898

[82] De Groot AS Moise L McMurry JA Wambre E Van Overtvelt L Moingeon P. Activation of natural regulatory T cells by IgG Fc-derived peptide “Tregitopes”. Blood. (2008) 112:3303–11. 10.1182/blood-2008-02-138073 18660382 PMC2569179

[83] De Groot AS Skowron G White JR Boyle C Richard G Serreze D. Therapeutic administration of tregitope-human albumin fusion with insulin peptides to promote antigen-specific adaptive tolerance induction. Sci Rep. (2019) 9:16103. 10.1038/s41598-019-52331-1 31695065 PMC6834854

[84] Pohar J Simon Q Fillatreau S. Antigen-specificity in the thymic development and peripheral activity of CD4(+)FOXP3(+) T regulatory cells. Front Immunol. (2018) 9:1701. 10.3389/fimmu.2018.01701 30083162 PMC6064734

[85] Prangtaworn P Chaisri U Seesuay W Mahasongkram K Onlamoon N Reamtong O. Tregitope-linked refined allergen vaccines for immunotherapy in cockroach allergy. Sci Rep. (2018) 8:15480. 10.1038/s41598-018-33680-9 30341299 PMC6195530

[86] Cousens LP Najafian N Mingozzi F Elyaman W Mazer B Moise L. *In vitro* and *in vivo* studies of IgG-derived Treg epitopes. (Tregitopes): a promising new tool for tolerance induction and treatment of autoimmunity. J Clin Immunol. (2013) 33(Suppl. 1):S43–9. 10.1007/s10875-012-9762-4 22941509 PMC3538121

[87] Sorde L Spindeldreher S Palmer E Karle A. Tregitopes and impaired antigen presentation: Drivers of the immunomodulatory effects of IVIg? Immun Inflamm Dis. (2017) 5:400–15. 10.1002/iid3.167 28560793 PMC5691310

[88] Jensen PEH Warnke C Ingenhoven K Piccoli L Gasis M Hermanrud C. Detection and kinetics of persistent neutralizing anti-interferon-beta antibodies in patients with multiple sclerosis. Results from the ABIRISK prospective cohort study. J Neuroimmunol. (2019) 326:19–27. 10.1016/j.jneuroim.2018.11.002 30447419

[89] Antonelli G Giannelli G Currenti M Simeoni E Del Vecchio S Maggi F. Antibodies to interferon (IFN) in hepatitis C patients relapsing while continuing recombinant IFN-alpha2 therapy. Clin Exp Immunol. (1996) 104:384–7. 10.1046/j.1365-2249.1996.43747.x 9099919 PMC2200453

[90] Prummer O. Treatment-induced antibodies to interleukin-2. Biotherapy. (1997) 10:15–24. 10.1007/BF02678213 9261546

[91] Ohashi PS Oehen S Buerki K Pircher H Ohashi CT Odermatt B. Ablation of “tolerance” and induction of diabetes by virus infection in viral antigen transgenic mice. Cell. (1991) 65:305–17. 10.1016/0092-8674(91)90164-T 1901764

[92] Tangri S Mothe BR Eisenbraun J Sidney J Southwood S Briggs K. Rationally engineered therapeutic proteins with reduced immunogenicity. J Immunol. (2005) 174:3187–96. 10.4049/jimmunol.174.6.3187 15749848

[93] McKoy JM Stonecash RE Cournoyer D Rossert J Nissenson AR Raisch DW. Epoetin-associated pure red cell aplasia: past, present, and future considerations. Transfusion. (2008) 48:1754–62. 10.1111/j.1537-2995.2008.01749.x 18482185 PMC2730535

[94] Mueller R Karle A Vogt A Kropshofer H Ross A Maeder K. Evaluation of the immuno-stimulatory potential of stopper extractables and leachables by using dendritic cells as readout. J Pharm Sci. (2009) 98:3548–61. 10.1002/jps.21672 19226629

[95] Azam A Gallais Y Mallart S Illiano S Duclos O Prades C. Healthy donors exhibit a CD4 T cell repertoire specific to the immunogenic human hormone H2-relaxin before injection. J Immunol. (2019) 202:3507–13. 10.4049/jimmunol.1800856 31101669

[96] Bozhinov A Handzhiyski Y Genov K Daskalovska V Niwa T Ivanov I. Advanced glycation end products contribute to the immunogenicity of IFN-beta pharmaceuticals. J Allergy Clin Immunol. (2012) 129:855–8 e856. 10.1016/j.jaci.2011.10.035 22154379

[97] Fan Y Rudert WA Grupillo M He J Sisino G Trucco M. Thymus-specific deletion of insulin induces autoimmune diabetes. Embo J. (2009) 28:2812–24. 10.1038/emboj.2009.212 19680229 PMC2750011

[98] Achenbach P Warncke K Reiter J Naserke HE Williams AJ Bingley PJ. Stratification of type 1 diabetes risk on the basis of islet autoantibody characteristics. Diabetes. (2004) 53:384–92. 10.2337/diabetes.53.2.384 14747289

[99] Ilonen J Hammais A Laine AP Lempainen J Vaarala O Veijola R. Patterns of beta-cell autoantibody appearance and genetic associations during the first years of life. Diabetes. (2013) 62:3636–40. 10.2337/db13-0300 23835325 PMC3781470

[100] Pathiraja V Kuehlich JP Campbell PD Krishnamurthy B Loudovaris T Coates PT. Proinsulin-specific, HLA-DQ8, and HLA-DQ8-transdimer-restricted CD4+ T cells infiltrate islets in type 1 diabetes. Diabetes. (2015) 64:172–82. 10.2337/db14-0858 25157096

[101] Babon JA DeNicola ME Blodgett DM Crevecoeur I Buttrick TS Maehr R. Analysis of self-antigen specificity of islet-infiltrating T cells from human donors with type 1 diabetes. Nat Med. (2016) 22:1482–7. 10.1038/nm.4203 27798614 PMC5140746

[102] So M Elso CM Tresoldi E Pakusch M Pathiraja V Wentworth JM. Proinsulin C-peptide is an autoantigen in people with type 1 diabetes. Proc Natl Acad Sci USA. (2018) 115:10732–7. 10.1073/pnas.1809208115 30275329 PMC6196477

[103] Nayak S Sivakumar R Cao O Daniell H Byrne BJ Herzog RW. Mapping the T helper cell response to acid alpha-glucosidase in Pompe mice. Mol Genet Metab. (2012) 106:189–95. 10.1016/j.ymgme.2012.03.009 22494547 PMC3574558

[104] Jones TD Phillips WJ Smith BJ Bamford CA Nayee PD Baglin TP. Identification and removal of a promiscuous CD4+ T cell epitope from the C1 domain of factor VIII. J Thromb Haemost. (2005) 3:991–1000. 10.1111/j.1538-7836.2005.01309.x 15869596

[105] James EA Kwok WW Ettinger RA Thompson AR Pratt KP. T-cell responses over time in a mild hemophilia A inhibitor subject: epitope identification and transient immunogenicity of the corresponding self-peptide. J Thromb Haemost. (2007) 5:2399–407. 10.1111/j.1538-7836.2007.02762.x 18034765

[106] James EA van Haren SD Ettinger RA Fijnvandraat K Liberman JA Kwok WW. T-cell responses in two unrelated hemophilia A inhibitor subjects include an epitope at the factor VIII R593C missense site. J Thromb Haemost. (2011) 9:689–99. 10.1111/j.1538-7836.2011.04202.x 21251204 PMC4323178

[107] Pandey GS Yanover C Miller-Jenkins LM Garfield S Cole SA Curran JE. Endogenous factor VIII synthesis from the intron 22-inverted F8 locus may modulate the immunogenicity of replacement therapy for hemophilia A. Nat Med. (2013) 19:1318–24. 10.1038/nm.3270 24037092 PMC4123441

[108] van Haren SD Herczenik E ten Brinke A Mertens K Voorberg J Meijer AB. HLA-DR-presented peptide repertoires derived from human monocyte-derived dendritic cells pulsed with blood coagulation factor VIII. Mol Cell Proteomics. (2011) 10:M110002246. 10.1074/mcp.M110.002246 21467215 PMC3108829

[109] Peyron I Hartholt RB Pedro-Cos L van Alphen F Brinke AT Lardy N. Comparative profiling of HLA-DR and HLA-DQ associated factor VIII peptides presented by monocyte-derived dendritic cells. Haematologica. (2018) 103:172–8. 10.3324/haematol.2017.175083 29025906 PMC5777204

[110] Jankowski W Park Y McGill J Maraskovsky E Hofmann M Diego VP. Peptides identified on monocyte-derived dendritic cells: a marker for clinical immunogenicity to FVIII products. Blood Adv. (2019) 3:1429–40. 10.1182/bloodadvances.2018030452 31053570 PMC6517663

[111] Dasgupta S Repesse Y Bayry J Navarrete AM Wootla B Delignat S. VWF protects FVIII from endocytosis by dendritic cells and subsequent presentation to immune effectors. Blood. (2007) 109:610–2. 10.1182/blood-2006-05-022756 16985172

[112] Meunier S Menier C Marcon E Lacroix-Desmazes S Maillere B. CD4 T cells specific for factor VIII are present at high frequency in healthy donors and comprise naive and memory cells. Blood Adv. (2017) 1:1842–7. 10.1182/bloodadvances.2017008706 29296830 PMC5728100

[113] Cannavo A Valsecchi C Garagiola I Palla R Mannucci PM Rosendaal FR. Nonneutralizing antibodies against factor VIII and risk of inhibitor development in severe hemophilia A. Blood. (2017) 129:1245–50. 10.1182/blood-2016-06-720086 28034891

[114] Theobald M Biggs J Hernandez J Lustgarten J Labadie C Sherman LA. Tolerance to p53 by A2.1-restricted cytotoxic T lymphocytes. J Exp Med. (1997) 185:833–41. 10.1084/jem.185.5.833 9120389 PMC2196170

[115] Kieback E Hilgenberg E Stervbo U Lampropoulou V Shen P Bunse M. Thymus-derived regulatory T cells are positively selected on natural self-antigen through cognate interactions of high functional avidity. Immunity. (2016) 44:1114–26. 10.1016/j.immuni.2016.04.018 27192577

[116] Miao CH Harmeling BR Ziegler SF Yen BC Torgerson T Chen L. CD4+FOXP3+ regulatory T cells confer long-term regulation of factor VIII-specific immune responses in plasmid-mediated gene therapy-treated hemophilia mice. Blood. (2009) 114:4034–44. 10.1182/blood-2009-06-228155 19713458 PMC2774545

[117] Costa-Pereira C Campi-Azevedo AC Coelho-Dos-Reis JG Peruhype-Magalhaes V Araujo MSS do Vale Antonelli LR. Multi-parameter approach to evaluate the timing of memory status after 17DD-YF primary vaccination. PLoS Negl Trop Dis. (2018) 12:e0006462. 10.1371/journal.pntd.0006462 29879134 PMC5991646

[118] Jutel M Akdis CA. Immunological mechanisms of allergen-specific immunotherapy. Allergy. (2011) 66:725–32. 10.1111/j.1398-9995.2011.02589.x 21466562

[119] Gueguen C Bouley J Moussu H Luce S Duchateau M Chamot-Rooke J. Changes in markers associated with dendritic cells driving the differentiation of either TH2 cells or regulatory T cells correlate with clinical benefit during allergen immunotherapy. J Allergy Clin Immunol. (2016) 137:545–58. 10.1016/j.jaci.2015.09.015 26522402

[120] Vultaggio A Nencini F Pratesi S Cammelli D Totaro M Romagnani S. IL-10-producing infliximab-specific T cells regulate the antidrug T cell response in exposed patients. J Immunol. (2017) 199:1283–9. 10.4049/jimmunol.1700008 28716826

[121] Akdis CA Blesken T Akdis M Wuthrich B Blaser K. Role of interleukin 10 in specific immunotherapy. J Clin Invest. (1998) 102:98–106. 10.1172/JCI2250 9649562 PMC509070

[122] Van Overtvelt L Wambre E Maillere B von Hofe E Louise A Balazuc AM. Assessment of Bet v 1-specific CD4+ T cell responses in allergic and nonallergic individuals using MHC class II peptide tetramers. J Immunol. (2008) 180:4514–22. 10.4049/jimmunol.180.7.4514 18354173

[123] Vita R Overton JA Greenbaum JA Ponomarenko J Clark JD Cantrell JR. The immune epitope database. (IEDB) 3.0. Nucleic Acids Res. (2015) 43:D405–12. 10.1093/nar/gku938 25300482 PMC4384014

[124] De Wit D Van Mechelen M Ryelandt M Figueiredo AC Abramowicz D Goldman M. The injection of deaggregated gamma globulins in adult mice induces antigen-specific unresponsiveness of T helper type 1 but not type 2 lymphocytes. J Exp Med. (1992) 175:9–14. 10.1084/jem.175.1.9 1370533 PMC2119093

[125] Aderem A Ulevitch RJ. Toll-like receptors in the induction of the innate immune response. Nature. (2000) 406:782–7. 10.1038/35021228 10963608

[126] Rombach-Riegraf V Karle AC Wolf B Sorde L Koepke S Gottlieb S. Aggregation of human recombinant monoclonal antibodies influences the capacity of dendritic cells to stimulate adaptive T-cell responses *in vitro*. PLoS ONE. (2014) 9:e86322. 10.1371/journal.pone.0086322 24466023 PMC3897673

[127] Ginsburg DS Floyd M Potter E del Greco F Levin ML. Tissue and glomerular deposition of globulin aggregates. J Lab Clin Med. (1981) 97:418–28. 6161975

[128] Verthelyi D Wang V. Trace levels of innate immune response modulating impurities (IIRMIs) synergize to break tolerance to therapeutic proteins. PLoS ONE. (2010) 5:e15252. 10.1371/journal.pone.0015252 21203556 PMC3008684

[129] Arnoult C Brachet G Cadena Castaneda D Azzopardi N Passot C Desvignes C. Crucial role for immune complexes but not FcRn in immunization against Anti-TNF-alpha antibodies after a single injection in mice. J Immunol. (2017) 199:418–24. 10.4049/jimmunol.1601246 28584008

[130] Gilliland LK Walsh LA Frewin MR Wise MP Tone M Hale G. Elimination of the immunogenicity of therapeutic antibodies. J Immunol. (1999) 162:3663–71. 10092828

[131] Yeung VP Chang J Miller J Barnett C Stickler M Harding FA. Elimination of an immunodominant CD4+ T cell epitope in human IFN-beta does not result in an *in vivo* response directed at the subdominant epitope. J Immunol. (2004) 172:6658–65. 10.4049/jimmunol.172.11.6658 15153481

[132] Mazor R Eberle JA Hu X Vassall AN Onda M Beers R. Recombinant immunotoxin for cancer treatment with low immunogenicity by identification and silencing of human T-cell epitopes. Proc Natl Acad Sci USA. (2014) 111:8571–6. 10.1073/pnas.1405153111 24799704 PMC4060717

[133] Holgate RG Weldon R Jones TD Baker MP. Characterisation of a novel anti-CD52 antibody with improved efficacy and reduced immunogenicity. PLoS ONE. (2015) 10:e0138123. 10.1371/journal.pone.0138123 26372145 PMC4570798

[134] Ettinger RA Liberman JA Gunasekera D Puranik K James EA Thompson AR. FVIII proteins with a modified immunodominant T-cell epitope exhibit reduced immunogenicity and normal FVIII activity. Blood Adv. (2018) 2:309–22. 10.1182/bloodadvances.2017013482 29444872 PMC5858479

[135] Mingozzi F High KA. Immune responses to AAV in clinical trials. Curr Gene Ther. (2011) 11:321–30. 10.2174/156652311796150354 21557723

[136] Wagner DL Amini L Wendering DJ Burkhardt LM Akyuz L Reinke P. High prevalence of *Streptococcus pyogenes* Cas9-reactive T cells within the adult human population. Nat Med. (2019) 25:242–8. 10.1038/s41591-018-0204-6 30374197

[137] Hosoya T Li H Ku CJ Wu Q Guan Y Engel JD. High-throughput single-cell sequencing of both TCR-beta alleles. J Immunol. (2018) 201:3465–70. 10.4049/jimmunol.1800774 30381480

[138] Rosati E Dowds CM Liaskou E Henriksen EKK Karlsen TH Franke A. Overview of methodologies for T-cell receptor repertoire analysis. BMC Biotechnol. (2017) 17:61. 10.1186/s12896-017-0379-9 28693542 PMC5504616

